# Dissecting wheat above-ground architecture for enhanced water use efficiency and grain yield in the subtropics

**DOI:** 10.1186/s40529-024-00419-x

**Published:** 2024-05-16

**Authors:** Sadia Hakeem, Zulfiqar Ali, Muhammad Abu Bakar Saddique, Muhammad Habib-ur-Rahman, Martin Wiehle

**Affiliations:** 1 Institute of Plant Breeding and Biotechnology, MNS University of Agriculture, Multan, Pakistan; 2 Department of Plant Breeding and Genetics, University of Agriculture, Faisalabad, Pakistan; 3 Programs and Projects Department, Islamic Organization for Food Security, Mangilik Yel Ave. 55/21 AIFC, Unit 4, C4.2, Astana, Republic of Kazakhstan; 4 Crop Science Group, Institute of Crop Science and Resource Conservation (INRES), University of Bonn, Bonn, Germany; 5 Organic Plant Production and Agroecosystems Research in the Tropics and Subtropics, University of Kassel, Steinstrasse 19, Witzenhausen, D-37213 Germany; 6 Centre for International Rural Development, University of Kassel, Steinstraße 19, Witzenhausen, D-37213, Germany

**Keywords:** Climate change, Drought, Heat stress, Leaf morphologys, *Triticale*, *Triticum durum*, *Triticum aestivum*, Scanning electron microscopy

## Abstract

**Background:**

Growing wheat under climate change scenarios challenges, scientists to develop drought and heat-tolerant genotypes. The adaptive traits should therefore be explored and engineered for this purpose. Thus, this study aimed to dissect surface traits and optimizing the leaf architecture to enhance water use efficiency (WUE) and grain yield. Twenty-six wheat genotypes were assessed for five novel leaf traits (NLTs: leaf prickle hairs, groove type, rolling, angle and wettability) under normal, drought and heat conditions following triplicated factorial randomized complete block design (RCBD). The data for NLTs, physiological traits (stomatal conductance, WUE, transpiration, and photosynthesis), and standard morphological and yield traits were recorded. Leaves were sampled at the stem elongation stage (Zadoks 34) to measure the leaf water content (%), contact angle, and to obtain pictures through scanning electron microscopy (SEM). The air moisture harvesting efficiency was evaluated for five selected genotypes. The ideotype concept was applied to evaluate the best-performing genotypes.

**Results:**

The correlation analysis indicated that long leaf prickle hairs (> 100 μm), short stomatal aperture and density (40–60 mm^− 2^), inward to spiral leaf rolling, medium leaf indentation, low contact angle hysteresis (< 10°), and cuticular wax were positively associated with WUE. This, in turn, was significantly correlated to grain yield. Thus, the genotypes (E-1) with these traits and alternate leaf wettability had maximum grain yield (502 g m^− 2^) and WUE supported with high photosynthesis rate, and relative water content (94 and 75% under normal and stress conditions, respectively). However, the genotype (1-hooded) with dense leaf hairs on edges but droopy leaves, spiral leaf rolling, and lighter groove, also performed better in terms of grain yield (450 g m^− 2^) under heat stress conditions by maintaining high photosynthesis and WUE with low stomatal conductance and transpiration rate.

**Conclusion:**

The SEM analysis verified that the density of hairs on the leaf surface and epicuticular wax contributes towards alternate wettability patterns thereby increasing the water-use efficiency and yield of the wheat plant. This study paves a way towards screening and and developing heat and drought-tolerant cultivars that are water-saving and climate-resilient.

**Supplementary Information:**

The online version contains supplementary material available at 10.1186/s40529-024-00419-x.

## Background

Current climate change scenarios including rise in temperature (1.5 °C above pre-industrial levels; Pörtner et al., 2022) and depleting water resources (20% reduction with each degree Celsius increase in temperature; Caretta et al. 2022), affect agricultural production. Wheat is the 2^nd^ most important cereal grown on an area of 219 million hectares with a global production of 808 million tons in 2022 (FAO 2024). Wheat consumption currently accounts for 50 kg capita^− 1^ year^− 1^ (averaged over 102 countries out of 173 wheat-growing countries; Erenstein et al. 2022; FAO 2024), while its production needs to be increased at an annual rate of 132 million metric tons (Erenstein et al. 2022). Although wheat is a C_3_ plant that benefits from a CO_2_ fertilization effect, a 1 to 3 °C rise in temperature counterweights this positive phenomenon (Janjua et al. 2014; Zhao et al. 2017). Past trends (1980–2010) indicated a decline in wheat yields by 5.5% due to 0.13 °C increase in decadal temperature (Lobell et al. 2011). Moreover, the increasing pressure of wheat demand, calls for more water reservoirs; wheat consumes about 1000 l of fresh water to produce 1 kg of grain (Erenstein et al. 2022) compared to maize (690 l), and barley (700 l) under subtropical conditions (Singh et al. 2014). Thus, the water shortage in developing countries may reduce wheat yields by 50–90% (Cochard et al. 2002).

The compound extreme events and increasing wheat demand, require the mandatory need of improved plant WUE. Plants exhibit multi-functional traits with intriguing surface structures developed over the past 350–450 million years (Barthlott et al. 2017). Crop plants can use these structures to withstand climate fluctuations by improving architecture and physiological mechanisms. The WUE is represented as CO_2_ assimilated biomass per unit of water utilized by the crop. The increasing CO_2_ concentration boosts WUE unless the leaf faces high-temperature stress (Hamim 2005). The response of leaf WUE is directly measured by physiological processes like vapor pressure deficit, transpiration rate, and stomatal conductance. A variety of methods are used to screen germplasm for the WUE under climate change scenarios (Hatfield and Dold 2019). At the leaf level, the WUE is governed by energy variables including vapor pressure deficit controlled itself by stomatal conductance, while the water balance in the soil in the form of evaporation and input also affects the WUE. Thus, leaf WUE dynamics must be considered at the canopy rather than individual leaf level considering the water input from soil and air. The WUE at the canopy level can be improved by either opting for agronomic management practices to reduce soil water evaporation like mulching, irrigation, management, and row spacing (Hatfield and Dold 2019) or via modification of plant architecture for early ground cover to reduce the evapotranspiration losses from the plant and the soil. For instance, leaf traits like leaf angle and rolling help wheat plants in adapting to drought and heat stress conditions (Merrium et al. 2022), while leaf area index (Zhang et al. 2021) and leaf anatomy characteristics like increased mesophyll cell conductance, reduced stomatal density, and increased cuticular wax, enhance the WUE (Gago et al. 2014). Overall, traits like photosynthesis and transpiration rate, crop growth pattern, and soil moisture content (M) must reflect the plant’s WUE.

The plant WUE can be achieved by two strategies: (i) exploiting and increasing plant WUE through genetics and breeding for plant architecture, (ii) improved agronomic practices to store water in the root zone of plants to reduce irrigation needs (Gago et al. 2014). Limited studies have been conducted to evaluate the impact of leaf architectural traits on WUE and these needs to be explored further. Therefore, this study aimed to assess the hypothesis that leaf wettability traits, contact angle hysteresis, as well as stomatal density and diameter may contribute to higher WUE and ultimately grain yield. These traits fall under the newly described term, Novel Leave Traits (NLTs – assembly of traits for air moisture channelling; Merrium et al. 2022). Five NLTs including leaf angle, leaf prickle hairs, groove type, leaf rolling, and leaf wettability were studied under normal, drought and heat conditions. The leaf abaxial and adaxial surface was also analysed for the leaf anatomical features like stomata, leaf hairs, and waxiness. Finally, an assembly of leaf surface traits was proposed for adaptation to combat climate stresses.

## Methods

### Climatic conditions

Climatic parameters including air and soil temperature (°C), relative humidity (%), wind speed (m s^− 1^), and rainfall (mm) were observed using an automatic weather station (CR1000X data logger, Campbell Scientific Inc., Logan, UT, USA) installed at 650–700 m distance from the experimental field (30°08’28” N, 71°26’73” E, 122 m) at MNS University of Agriculture, Multan-Pakistan. The weather variables were logged at 15 min intervals throughout the growing season (15 November 2020 to 14 April 2021). The means of maximum, minimum, and average air temperature were 25, 13 and 19 °C, respectively (Figure S1a). The average seasonal relative humidity at 04:00, 14:00 and 20:00 h was 84, 46 and 71%, respectively; highest during December and January (93–94%) and lowest in April (60%; Figure S1b). The average precipitation was 0.004 mm indicative of no rainfall throughout this season (Figure S2a). A total of eight fog events occurred with an average visibility range of less than 1000 m and relative humidity of more than 90% (Figure S2b).

### Plant material and experimental design

Twenty-six wheat genotypes (including 16 *Triticum aestivum*, 7 *Triticum durum*, and 3 Triticosecale (*Triticale*); Table S1) were sown at a rate of 45–60 plants m^− 2^ following factorial randomized complete block design with three replications, each for normal (3 irrigations - tillering, booting and grain filling stages), drought (2 irrigations - tillering and booting stages only) and heat stress conditions (sown 40 days after normal sowing,  ± 5 °C). The row-to-row distance was 16 cm, and the harvested plot area was 1 m^2^. Di-ammonium phosphate and sulfate of potash were applied as a basal dose at the rate of 50 kg ha^− 1^. Recommended management practices for wheat in central Punjab were followed (AARI 2019). The experiment was repeated in the next growing season (17 November 2021 to 14 April 2022) to evaluate the genotypes for leaf contact angle (CA), relative water content (RWC), and their epidermal structures through scanning electron microscopy (SEM). Five leaf samples per genotype and replication (390 total samples) were collected for CA, RWC, and SEM.

### Phenotyping

#### Novel leaf and other phenological traits

Genotypes were evaluated for five novel leaf traits viz. prickle hairs (PH), groove type (Hakeem et al., 2021), leaf angle (LA), rolling (Merrium et al. 2022) and contact angle (CA) at the stem elongation stage (Zadoks 34). The leaf contact angle as a measure of surface wettability was recorded using the OCA-25 Contact Angle Device (Data-Physics Instruments, Filderstadt, Germany) at room temperature (26–30 °C, 49–69% relative humidity). The Static Contact Angle (SCA) and Contact Angle Hysteresis (CAH) were measured following Huhtamäki et al. (2018). Based on SCA data, six genotypes with contrasting leaf and wettability features were selected to measure the CAH. Three readings each were taken at the base, mid and tip portion of the abaxial and adaxial leaf surface (eighteen readings per genotype). Other phenological traits including whole plant traits (productive tillers per plant, plant height, days to heading and maturity), flag leaf characters (twist, erectness, length, width, and area), stem characteristics (diameter, wall thickness, peduncle length, and lodging percentage), spike characteristics (spikelet per spike, length, number of seeds per spike, and spike weight), and grain yield per plot (g) were recorded at appropriate stages throughout the growing season.

#### Physiological parameters

Physiological traits including photosynthesis rate (P; µmol CO_2_ m^− 2^ s^− 1^), stomatal conductance (gs; mmol H_2_O m^− 2^s^− 1^), transpiration rate (E; mmol H_2_O m^− 2^s^− 1^), and photosynthetic WUE (mmol CO_2_ mol^− 1^ H_2_O) were recorded at the stem elongation stage (Zadoks 34). The data were recorded using the CIRAS-3 Portable Photosynthesis System (PP Systems, Amesbury, MA, USA) between 12:00 am and 03:00 pm. The expanded portion of the leaf blade was used to record the data. Three readings taken for each genotype per replication were averaged. The intrinsic WUE (WUE_int_) was calculated as ratio of P and gs while instantaneous WUE (WUE_inst_) was calculated as ratio of P and T (Hatfield and Dold 2019) to account for the long-term and short-term efficiency of the wheat plants, respectively. The leaf RWC was calculated using the formula [(FW-DW)/(TW-DW)] × 100, where FW, DW and TW represent fresh, dry and turgid weight of the leaf tissues, respectively. The fully grown fresh leaf samples (2^nd^ leaf from the top) collected at the stem elongation stage were weighed for FW, TW (saturated in distilled water, usually for 24 h until full turgidity), and DW (dried in an oven at 65 °C until constant weight). The experiment was conducted in three replicates and the average was calculated.

#### Measurement of stem flow water

To measure stem flow water (SFW), the collector designed following Ebner et al. (2011) was attached to the plant’s base in the afternoon (04:00 pm) and sampled the next morning between 08:00–09:00 am, consecutively for three foggy days for each fog event.

#### Scanning electron microscopy analysis for leaf surface structure

Leaf surface structures were studied using Raster Scanning Electron Microscopy (S-4000, Hitachi Global, Tokyo, Japan) available at the Institute of Chemistry, University of Kassel, Germany. The dried leaf cross-sections were mounted on stubs and sputtered with platinum (12 nm, Model SCD 005/CEA 035, BAL-TEC GmbH, Witten, Germany). The specimen was observed under vacuum, with an accelerating voltage of 10 kV, and a working distance of 15 mm.

#### Statistical analysis and data display

All statistics were performed with R software v. 4.1.2 (R Core Team, 2020). The analysis of variance was performed using the *agricolae* package. For the evaluation and selection of genotypes for each trait, a biplot was plotted using package *ggbiplot2* utilizing the scaling and centring features of the package. The relationship among traits was developed using Pearson’s correlation (package *corrplot*). Graphs, including radar plots to display the performance of investigated genotypes to serve the ideotype approach, and heatmaps were Excel generated. Conditional formatting was applied for the heat map with green representing the desired trait while red for the undesirable trait. The SEM images were analysed using ImageJ version 1.52 (Schneider et al. 2012). An area of 0.56 mm^2^ was used per picture to calculate the stomatal and PH densities on the leaf surfaces and was estimated for 1 mm^2^ using the formula (n × (1/0.56), n being the number of stomata/PH per picture).

## Results

### Phenotypic response of genotypes under normal, drought and heat stress conditions

**Fig. 1:**
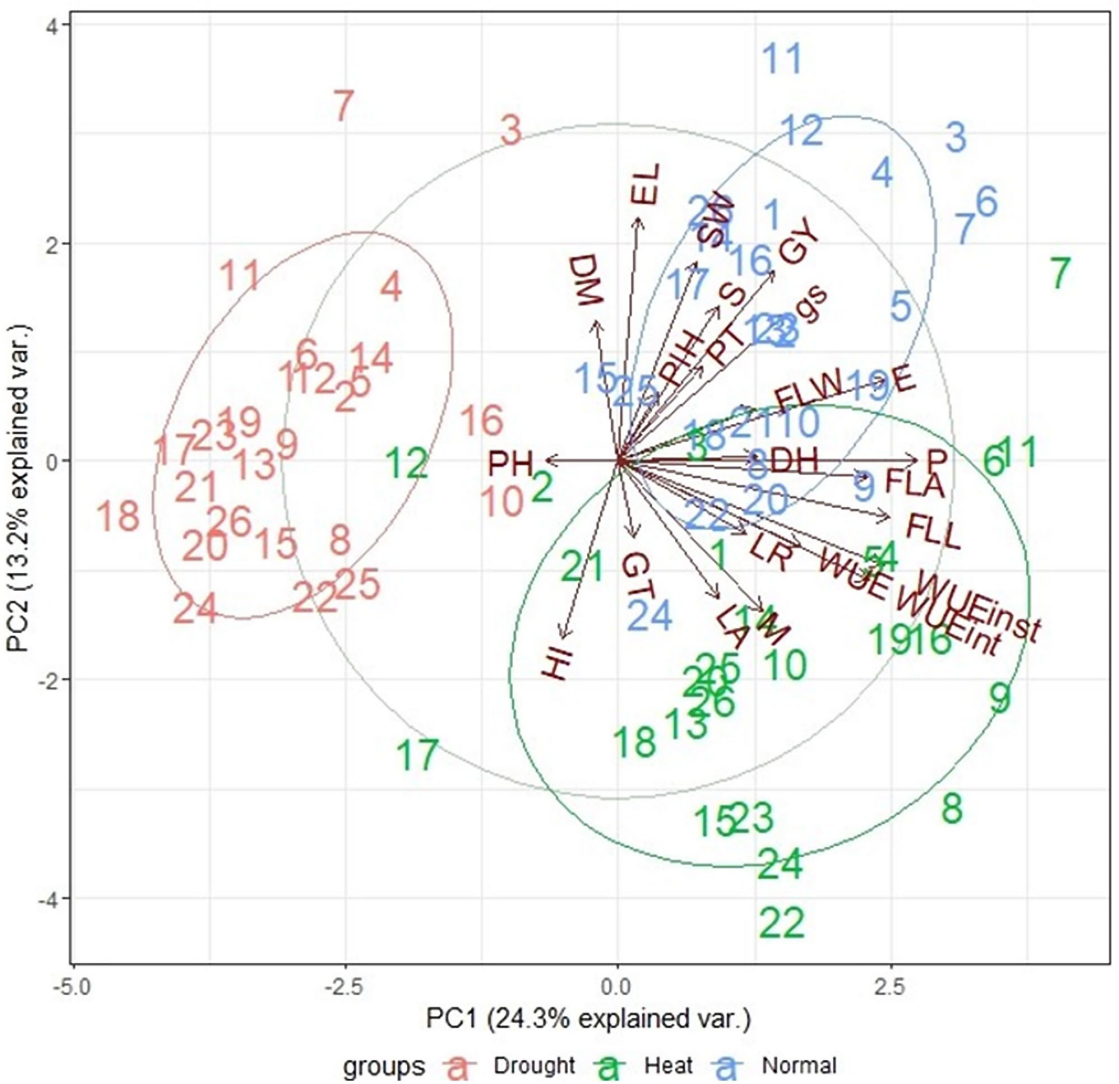
Genotype-trait-environment biplot analysis of the 26 wheat genotypes for the novel leaf traits (NLTs), morpho-physiological and yield traits under normal, drought and heat stress conditions. LA: leaf angle, PH: prickle hairs, GT: groove type, LR: leaf rolling, FLL: flag leaf length (cm), FLW: flag leaf width (cm), FLA: flag leaf area (cm^2^), EL: ear length (cm), PlH: plant height (cm), DH: days to heading, PT: productive tillers, DM: days to maturity, M: soil moisture content, gs: stomatal conductance (mmol H_2_O m^− 2^ s^− 1^), P: photosynthesis (µmol CO_2_ m^− 2^ s^− 1^), E: transpiration (mmol H_2_O m^− 2^ s^− 1^), WUE: photosynthetic water use efficiency (mmol CO_2_ mol^− 1^ H_2_O), WUE_inst_: instantaneous water use efficiency, WUE_int_: intrinsic water use efficiency, SW: seed weight per spike (g), HI: harvest index per plot (%), S: number of seeds per ear, GY: grain yield per plot (g)

The analysis of variance for the phenotypic data indicated significant variation (*p < 0.001*) for all the traits, genotypes, treatments, and their interaction (G×E; Table S2, S3, and S4) except for a few yield-related traits (spike length - EL, days to maturity - DM, grain filling duration - GFD, seed weight per spike - SW, and harvest index per spike - HI/spike) where G×E interaction was non-significant (Table S3, and S5). The 23.9% and 13.9% variation of the genotype-trait-environment biplot for the morpho-physiological traits of 26 wheat genotypes was explained by PC1 and PC2, respectively. Generally, genotypes showed higher yield-contributing traits under normal conditions while high P, WUE, LR, and LA under heat stress conditions and maximum PH under drought conditions. Thus, genotypes were clustered according to these adaptive traits under normal, drought and heat stress conditions (Fig. 1). Under normal condition, genotypes coded as 3, 4, 5, 6, 7, 11, and 12 had highest scores for yield-contributing traits including grain yield per plot (GY), productive tillers (PT), SW, EL, and number of seeds per spike (S) (Figure S3a). Under drought conditions, genotype 11 followed by 12 had the highest GY, WUE and GT in contrast to genotype 10 having lowest score for these traits (Figure S3c). Under heat stress, genotypes 7 and 11 had higher P, SW, S, and GY. Genotype 9 had the highest WUE while genotypes 22 and 24 had the highest NLTs (Figure S3e).

The WUE_inst_ and WUE_int_ had a significant negative association with the LR, but a significant positive association with GY (Figure S3b, S4). The GY and other yield traits were significantly positively related to physiological parameters like stomatal conductance, transpiration and P under all treatments (Figure S3b, d, f). The M, however, had a negative association with WUE under drought and heat conditions (Figure S3d, f).

### Leaf contact angle dynamics, relative water content, and water use efficiency

**Fig. 2:**
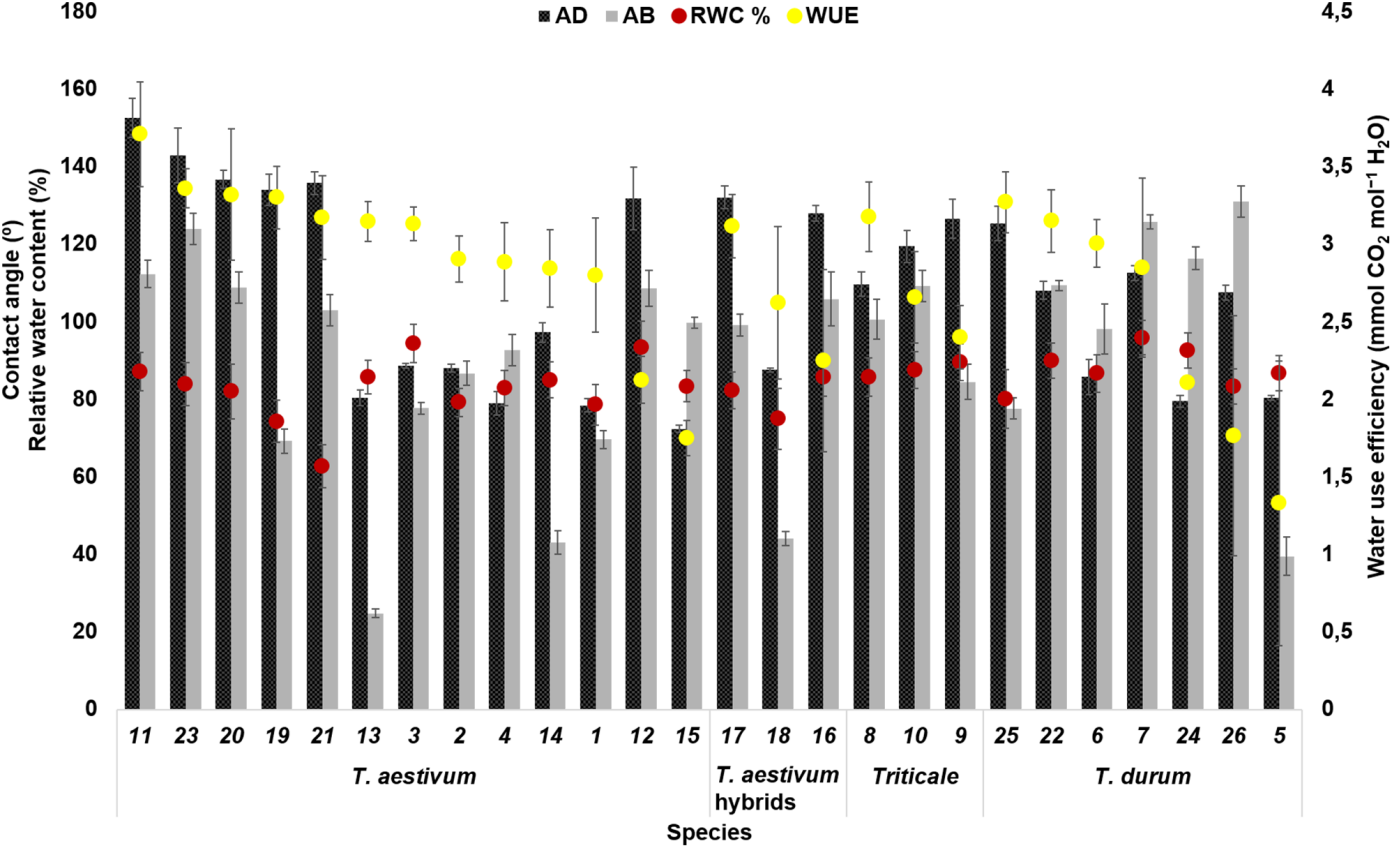
Static contact angle on the abaxial and adaxial leaf surface and leaf relative water content of 26 wheat genotypes. AD: contact angle on the adaxial leaf surface (°), AB: contact angle on the abaxial leaf surface (°), RWC: leaf relative water content (%); results are presented as mean ± standard deviation

Fourteen genotypes had leaf RWC > 80%, eight genotypes had < 80%, while only four genotypes (coded as 3, 7, 12, 24) had > 90% (Fig. 2). Among species, the average WUE was highest for *T. aestivum* preceded by *Triticale* and lowest for *T. durum* while RWC and CA were higher for *Triticale* and durum compared with bread wheat. The wettability (CA < 90°) were positively associated with WUE and GY (Figure S4). The RWC showed a significantly positive correlation with WUE, GT and a negative association with LR. Based on the SCA, genotypes were categorized into four different categories:

1. > 90° for both abaxial and adaxial surface (*n* = 12).
2. > 90° on adaxial surface and < 90° on abaxial surface (*n* = 5).
3. < 90° on adaxial surface and > 90° on abaxial surface (*n* = 5).
4. < 90° on both surfaces (*n* = 4).

Overall, the majority of genotypes (*n* = 22) had hydrophobic contact angle (i.e., > 90°) on the adaxial or abaxial leaf surface.

**Fig. 3:**
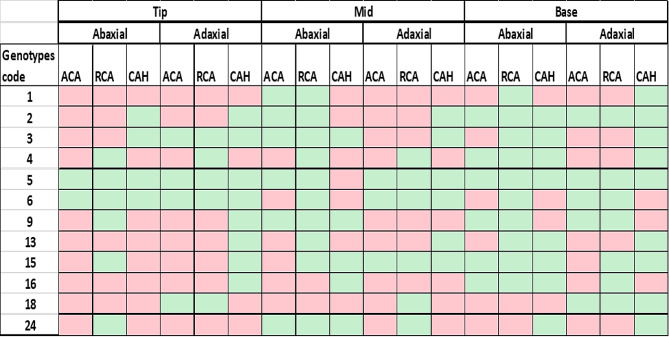
Heat map presenting the contact angle dynamics for the different leaf portions of abaxial and adaxial leaf surfaces of 12 selected wheat genotypes. The red color indicates cell values greater than 90° for Advancing Contact Angle (ACA) and Receding Contact Angle (RCA) and 10° for CAH, while green values indicate less than 90° for ACA and RCA and 10° for CAH. ACA: advancing contact angle, RCA: receding contact angle, CAH: contact angle hysteresis

Twelve genotypes from the above-mentioned groups were used to evaluate dynamics of CAH across the abaxial and adaxial leaf surfaces. The results indicated very heterogeneous wetting behaviour throughout the surface i.e., tip, middle and base of the leaf surface. Genotypes 1 and 5 showed contrasting behaviour i.e., the latter being hydrophilic (< 90°) and highly drop rolling efficien throughout the leaf surface for abaxial as well as adaxial surface. Genotypes coded as 9, 13, 15, 16, 18 and 24 showed similar behaviour being hydrophobic (CA > 90°) on the tip surface of adaxial and abaxial leaf. Genotype 13 was hydrophilic mostly on mid and base surfaces contrasting with genotype 18 (Fig. 3). Genotype 3 showed minimum CAH regardless of the nature of leaf surface. Overall, the CAH was favourable (< 10°) for drop rolling at the adaxial surface for most of the genotypes (2, 3, 5, 13 and 15).

**Fig. 4:**
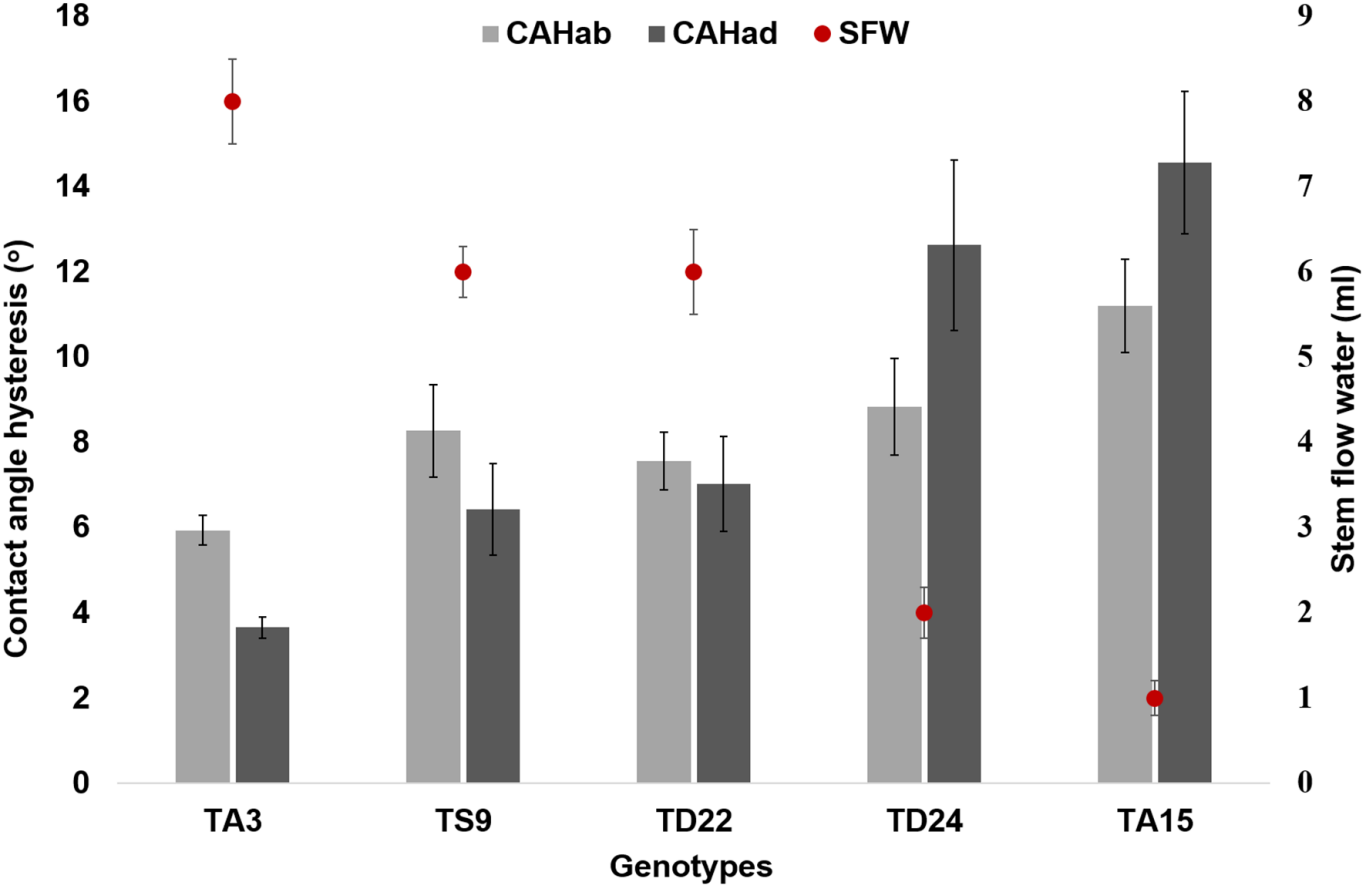
Average water budget measured as a result of stem flow and contact angle hysteresis of abaxial and adaxial leaf surface for five selected wheat genotypes in natural fog events under field conditions. SFW: stem flow water, CAHad: contact angle hysteresis for adaxial leaf surface, TA: *Triticum aestivum,* TD: *Triticum durum*; TS: *Triticale;* results are presented as mean ± standard deviation

Five genotypes with contrasting leaf architecture and contact angle properties were used to evaluate the air moisture capturing efficiency (Fig. 4). The total air moisture capturing ranged from 1 to 8 ml for different genotypes depending upon drop rolling efficiency indicated by CAH. The genotypes with lower CAH had higher SFW, for example, genotype 3. In contrast, genotype 15 with highest CAH had lowest SFW (1 ml).

### Surface structure of wheat leaves

**Fig. 5:**
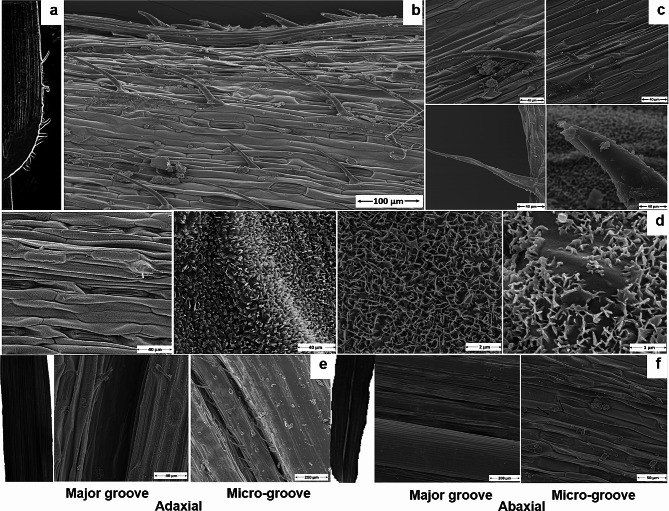
Leaf surface structures found on wheat leaf under Scanning Electron Microscope (SEM). **(a)** Leaf prickle hairs observable with naked eye. **(b)** Leaf hairs on the edges of leaf tips under SEM. **(c)** Different prickle hairs are found on adaxial leaf surface. **(d)** Waxy cuticular layer observed at different magnifications on adaxial leaf surface **(e)** Leaf groove observed from middle portion of the adaxial leaf surface. **(f)** Leaf groove observed from middle portion of the abaxial leaf surface with micro channels presented as black arrows

**Fig. 6:**
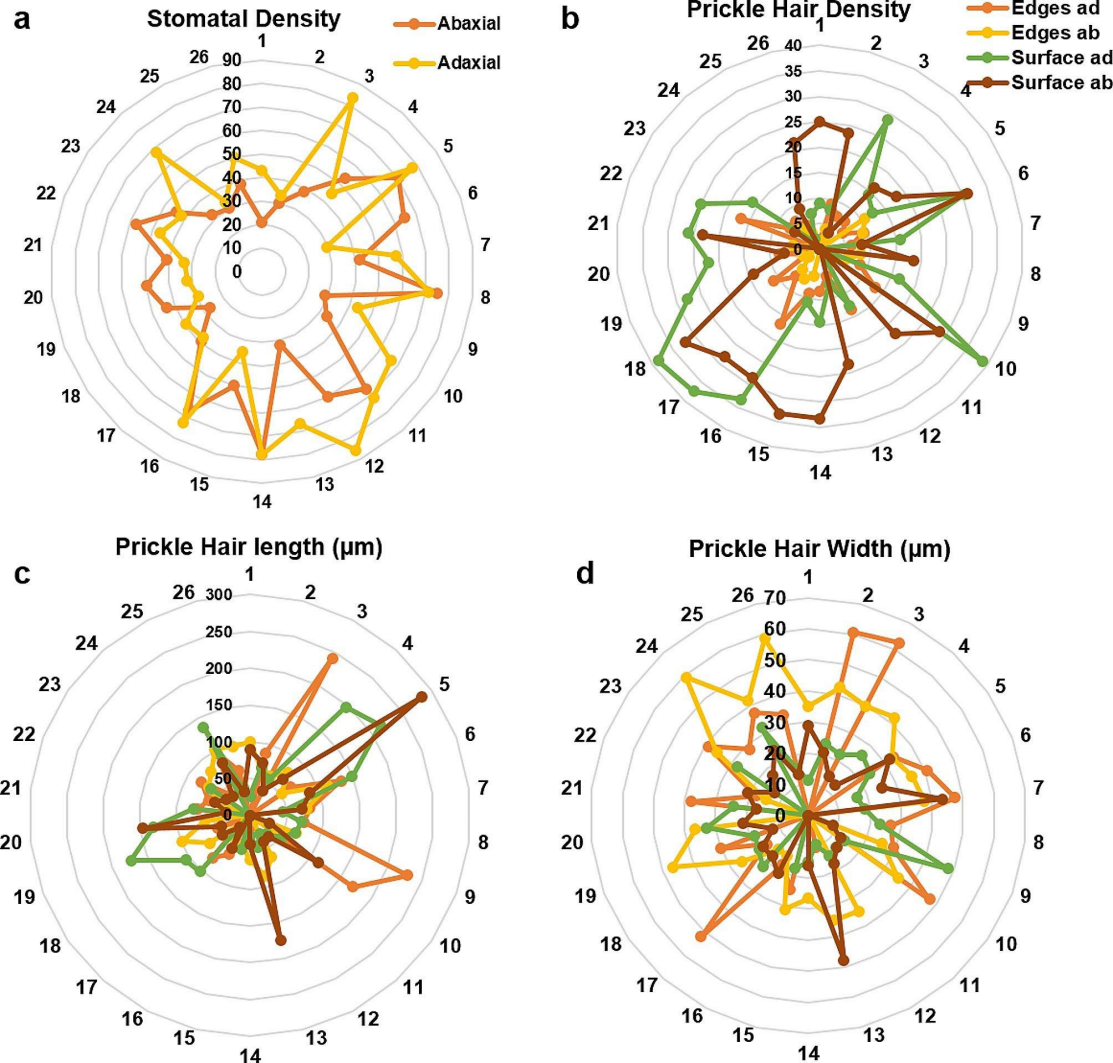
Characteristics of leaf surface structures for 26 wheat genotypes. **(a)** Density of stomata on the abaxial and adaxial leaf surface. **(b)** Density of prickle hairs on the edges, and abaxial and adaxial surface of leaf. **(c)** Prickle hair length on the edges, and abaxial and adaxial surface of leaf. **(d)** Prickle hair length on the edges, and ﻿abaxial and adaxial surface of leaf. ad: adaxial leaf surface, ab: abaxial leaf surface. The density of stomata and leaf were calculated for mm^2^ area

A marked feature of the wheat leaf is the presence of prickle hairs (Fig. 5a-c), waxy layer (Fig. 5d), longitudinal grooves/ridges, and microgrooves along the leaf surface (Fig. 5d-e). The major groove had a diameter of 162 ± 59 μm on different portions of abaxial surface while it was 67 ± 20 μm on the adaxial leaf surface. The microgroove had 50 ± 20 μm diameter. The stomatal diameter was 49 ± 14 μm (Fig. 5b). The trichomes were as large as 952 μm in length at base surface (found on adaxial basal portion of genotype 1, Fig. 6c) and as short as 7 μm (found on basal portion of adaxial leaf surface of genotype 17). However, only non-glandular trichomes were found on wheat leaves. Spiky/prickle hairs were also found alongside the length of microgrooves in some genotypes (Fig. 5e). The wax layer covered the leaf surface in a regular pattern especially abaxial surface in most of the genotypes and hairs may or may not be covered by this wax (Fig. 5b and c).

The stomatal density ranged from 21 to 85 stomata per mm^2^, maximum on adaxial surface for genotype 12 while minimum for genotype 1 on abaxial surface (Fig. 6a). The PH ranged from 0 to 38 per mm^2^, highest for genotype 10 and 18 on adaxial surface (Fig. 6b). The PH length was highest for genotype 5 and lowest for genotype 17 (Fig. 6c) while the PH width was highest for genotype 3 (62 μm) on abaxial edges and lowest for genotype 18 (10 μm; Fig. 6d). Generally, stomatal and prickle hair density and cuticular wax were found higher at adaxial surface than the abaxial surface.

## Discussion

Breeding high-yielding and water-saving genotypes is proposed to identify heat and drought tolerant germplasm for crop improvement programs (Hatfield and Dold 2019). The current study determined water use efficient wheat genotypes under normal, drought and heat conditions. The analysis of variance indicated mostly significant variations among genotypes, treatments, and G×T interactions, a result similarly confirmed by Siahpoosh et al. (2011) for physiological traits like WUE, gs, and E under normal and drought conditions. This variation among genotypes can thus be harnessed for climate adaptability. The genotype-trait-environment (GGE) biplot analysis was used to select genotypes, as G×E is an important predictor of genotype adaptability to environment (Kelly 2019). Genotypes with lowest scale of NLTs but higher values for WUE and yield-related characters were considered best performing genotypes. For example, the genotypes 3, 7 and 11 with high GY, S, and SW, but lowest values of HI, LA, PH, LR, and GT can be considered best performing for yield and adaptational traits, respectively (Fig. 1). Overall, under normal, drought and heat stress conditions, genotypes 3, 7, 11, 12 had highest GY, P, WUE, S, SW, and lowest PH, and were early maturing (minimum DH), had spiral LR and moderate E and gs (Figure S3) indicating the resilience of these genotypes under arid conditions featured with drought and heat stress tolerance (Bhandari et al. 2022).

Improving plant WUE in terms of intrinsic and instantaneous WUE and water diversion into the root zone are gaining attention for improved yield and climate resilience (Gago et al. 2014). However, WUE is a complex trait and genetic screening for plants with improved leaf and canopy traits is a prerequisite for improved WUE (Peng and Krieg 1992). Three of the NLTs (LA, LR, GT) showed highest scores on scale under heat stress condition i.e., genotypes had droopy leaf angles, outward to spiral rolling, and lighter leaf grooves (Fig. 1). The flattened leaves in this condition, however, were considered as an indicator for water saving (Huang et al. 2023). The canopy architecture of stress tolerant genotypes featured semi-droopy to droopy leaf angles, medium groove and spiral leaf rolling under all conditions. The comparison of different *Triticum* species indicated that *T. durum* genotypes (genotypes 8, 9, 25, and 26) had comparatively higher WUE (> 4.00 mmol CO_2_ mol^− 1^ H_2_O) under heat and drought stress conditions (Figure S4) supporting its higher adaptability under dry environments (Roques et al. 2016). Contrarily, under normal conditions, WUE was found higher for *T. aestivum* compared with *T. durum* (Fig. 2). The P was highest for *Triticale* preceded by durum and bread wheat supported by a previous study (Méndez-Espinoza et al. 2019). *Triticale* genotypes showed consistent WUE under all conditions (Figure S4) indicating a high resilience (Blum 2014) probably due to higher wax density evenly distributed across the leaf surface compared with bread wheat and durum wheat (Fig. 6).

Leaf traits, however, were influenced by environmental conditions and showed variable responses similar to studies by Niklas et al. (2023) and An et al. (2021), except for groove type in our study (Fig. 1, Fig. S5, Table S2). The dense PH was prominent on adaxial surface and edges of leaf under heat stress (Fig. S6). Wang et al. (2021) supported that density of trichomes increases under high temperature, contributing to high WUE (Xiao et al. 2013). The SEM analysis also indicated that these genotypes (G5, for instance) had long PH on surface (283 μm), moderate PH density and lower stomatal density (26–30 and 28–40 per mm^2^, respectively). Variations among genotypes for these traits further elucidated the leaf surface structures to support contrasting behaviour of genotypes for air moisture capturing and drought tolerance. Leaf grooves width ranged from 50 to 162 μm, PH from 24 to 952 μm and stomatal diameters from 38 to 81 μm. Higher variation among genotypes for trichome density, length, width, stomatal density, and aperture were found (Fig. 6) as also supported by Liao et al. (2005). One-third (9 out of 26) of the genotypes had less than 40 stomata per mm^2^ and only genotype 12 had as high as 85 stomata per mm^2^. The physiological parameters like gs, E, and WUE of genotypes also varied with these surface properties: for instance, genotype 10 showed minimum WUE (1.13 mmol CO_2_ mol^− 1^ H_2_O) as this genotype had higher stomatal conductance (high stomatal density-33/66 on abaxial/adaxial leaf surface per mm^2^) and E (even though PH density on surface was high-37 per mm^2^) (Huang et al. 2023). Genotypes with lower hair densities under stressed conditions (genotype 1, for instance) could, however, not perform better under drought and normal conditions because they had higher E, gs, SD, and P. Rather, the genotypes with semi-erect leaf angle, dense hair on edges but light on surface, and medium leaf groove supporting inward rolling (for example, genotype 3, 4) performed superior in terms of yield and WUE under normal and stressed conditions. Also, they captured maximum amount of moisture (8 ml) as compared with genotype 24 with droopy LA, no PH, medium leaf grooves and outward LR (Figure S7), and minimum stem flow water (1 ml). Martorell and Ezcurra (2007) supported that narrow leaves with grooves are efficient in capturing air moisture and to reduce water losses. These results are in line with a study on *Stipagrostis sabulicola* (Roth-Nebelsick et al. 2012). However, genotypes 14 and 20 performed best only under drought stress with similar leaf characteristics as of genotypes 3, 4, but managed to reduce water losses by almost cutting off gs and E (the SD was high, but the genotypes managed to close stomata under drought) and thus increasing WUE and GY. While, under heat stress condition, genotype 7 followed by 24 had highest GY and WUE with semi-droopy to droopy leaf angle, low SD, E and gs. The PH was wider at the base (18 ± 7 μm) and narrowed down towards the tip reaching 3 ± 2 μm in diameter. The longer and slender (narrow at the base) these were, the better their air moisture capturing and diversion effect (Ebner et al. 2011). Moreover, the stomatal and PH densities were typically found higher on the adaxial leaf surface of all the species as also supported by Wall et al. (2022). The density of stomata in the current study (< 85 mm^− 2^) is comparable to the ideotype of wheat for low rainfall environments under field conditions (Shahinnia et al. 2016), thus indicating the genotypes’ resilience for drought tolerance. The cuticular wax with microcrystalline structures was found instead of wax platelets as in *Stipagrostis sabulicola* grass (Fig. 5d; Roth-Nebelsick et al. 2012).

The bread wheat genotype had lower CA values (tendency to hydrophilicity) compared with other species (Fig. 2). Contrary to previous results where hydrophilicity was found to be more efficient for air moisture harvesting (Hakeem et al. 2023), the drop rolling efficiency presented in our study as CAH was found to be crucial for stem flow irrespective of the hydrophobicity or hydrophilicity character of the leaf surface. Overall, the larger CAH indicated non-wettable surface while lower CAH represented wettable leaf structure (Behnoudfar et al. 2022). The PH in the micro-groove structures of leaves may also facilitate water droplet movement (Fig. 6e). The negative association of WUE with LA and LR and positive association with RWC and GY (Zhang et al. 2010) supports that the genotypes with assembly of aforementioned traits had higher grain yield (Figure S5) possibly due to enhanced light interception, canopy cooling, and heat avoidance (Acreche and Slafer 2011). Thus, it can be concluded that differential manipulation of leaf angle throughout canopy such that upper leaves have relatively lower leaf angles (30–45°, upright stature) to facilitate air moisture interception and light penetration while droopy leaf angle (65–90°, loose architecture) for lower leaves to retain water and reduce water losses (Mantilla-Perez and Salas Fernandez 2017). This concludes that phenotypic plasticity is more important for climate resilience than the individual response of genotypes under optimized or single stress conditions. Finally, it should be mentioned that especially plant hairiness is not only a means to reduce water loss, but also an effective frost protection, which has been much better studied in herbs (Gorb and Gorb 2022). Under changing climatic conditions, cold extremes may also be expected in tropical and subtropical regions. Improved varieties with NLTs to increase WUE could therefore serve dual purposes under climate change conditions.

### Conclusions

The erratic climate patterns demand phenotypic plasticity of wheat to adapt increasing heat and drought episodes. An ideotype with differential leaf canopy for light interception, improved photosynthesis, lower contact angle hysteresis to facilitate air moisture diversion into the rootzone, longer prickle hairs on leaf surface, lower stomatal density can be a strategic approach to enhance water use efficiency in grasses like wheat. Five genotypes were found to have higher grain yield and water use efficiency under a stressed environment with varying functional traits. Genotype E-1 with semi-erect leaf angle supporting inward rolling under normal and drought conditions but semi-droopy leaves with spiral rolling under heat stress, dense hair on edges but light on surface, performed superior in terms of yield and WUE under normal and stress conditions. These genotypes could then be targeted to develop ideotypes and varieties for drought and heat tolerance.

### Electronic supplementary material

Below is the link to the electronic supplementary material.

Supplementary Material 1

## Data Availability

All data generated or analysed during this study are included in this published article [and its supplementary information files]. The raw data files and methods are available from the corresponding author upon reasonable request.

## Abbreviations

ACA: Advancing contact angle (°)
CA: Contact angle (°)
CAH: Contact angle hysteresis (°)
DH: Days to heading
DM: Days to maturity
EL: Spike length (cm)
gs: Stomatal conductance (mmol H_2_O m^− 2^ s^− 1^)
GFD: Grain filling duration (days)
GT: Groove type
GY: Grain yield per plot (g)
HI/spike: Harvest index per spike
LA: Leaf angle
LR: Leaf rolling
LW: Leaf wettability
M: Soil moisture content (%)
NLTs: Novel leaf traits
P: Photosynthesis rate (µmol CO_2_ m^− 2^ s^− 1^)
PH: Prickle hairs
PT: Productive tillers (PT)
RCA: Receding contact angle (°)
RWC: Leaf relative water content (%)
SFW: Stem flow water (ml)
SCA: Static contact angle (°)
SW: Seed weight per spike (g)
S: Number of seeds per spike
SEM: Scanning electron microscope
E: Transpiration rate (mmol H_2_O m^− 2^ s^− 1^)
WUE: Photosynthetic water use efficiency (mmol CO_2_ mol^− 1^ H_2_O)
WUE_int_: Intrinsic WUE (µmol CO_2_ m^− 2^ s^− 1^/ mmol H_2_O m^− 2^ s^− 1^)
WUE_inst_: Instantaneous WUE (µmol CO_2_ m^− 2^ s^− 1^/ mmol H_2_O m^− 2^ s^− 1^)
