# Supplementary material for: Dissecting wheat above-ground architecture for enhanced water use efficiency and grain yield in the subtropics
Source: Bot Stud. 2024 May 16;65:13. doi: 10.1186/s40529-024-00419-x (PMC11098988; doi:10.1186/s40529-024-00419-x)
Supplement: Supplementary file 1 — Supplementary Material 1 [file 40529_2024_419_MOESM1_ESM.docx]

**Dissecting wheat above-ground architecture for enhanced water use efficiency and grain yield in the subtropics**

Sadia Hakeem^1^, Zulfiqar Ali*,^1,2,3^, Muhammad Abu Bakar Saddique^1^, Muhammad Habib-ur-Rahman^1,4^, Martin Wiehle*,^5,6^

^1^Institute of Plant Breeding and Biotechnology, MNS University of Agriculture, Multan, Pakistan

^2^ Department of Plant Breeding and Genetics, University of Agriculture, Faisalabad, Pakistan

^3^ Programs and Projects Department, Islamic Organization for Food Security, Mangilik Yel Ave. 55/21 AIFC, Unit 4, C4.2, Astana, Republic of Kazakhstan

^4^Institute of Crop Science and Resource Conservation (INRES), Crop Science Group, University of Bonn, Germany

^5^Organic Plant Production and Agroecosystems Research in the Tropics and Subtropics, University of Kassel, Steinstrasse 19, D-37213 Witzenhausen, Germany

^6^Centre for International Rural Development

* Corresponding authors: [zulfiqarpbg@hotmail.com](mailto:zulfiqarpbg@hotmail.com), [wiehle@uni-kassel.de](mailto:wiehle@uni-kassel.de)

**Table S1:** Coreset of 26 genotypes including bread wheat, durum wheat and triticale used in this study

| **Species** | **Genotype code** | **Genotype** | **Pedigree** | **Features** |
| --- | --- | --- | --- | --- |
| *T. aestivum* | 25 | GA 387 | PVN//CAR422/JAZ2*2/… | Drought tolerant |
|  | 65 | B-9 | Aus-7-56-0806//PBI0036 | Air-moisture capturing |
|  | 87 | E-1 | ATILA/3*BCN//BAV92/3/PASTOR/4/ | Heat tolerant |
|  | 88 | 25SAWYTE305 | PSN/BOW//SERI/3/MILAN/4/ATILLA.5/  KAUZ*2/CHEN//BCN/3/… | Heat tolerant |
|  | 100 | Bakhar Star |  | High yielding cultivar |
|  | 64 | Zincol | OASIS/SKAUZ//4*BCN/3/2*PASTOR/4/T. SPELTA PI348449/5/BACEU#1/6/WBLL1*2/CHAPIO | Zn fortified cultivar |
|  | 301 | SD 4 (866) | 29SAWSN11-12/40 | Air-moisture capturing but low yield |
|  | 302 | SD 6 (253) | AUS-12-1028 × PBI09C048-BC-0C-6N-99N //AUS-12-1028 | High yielding |
|  | 305 | SD 9 (22) | Aus-7-64-0971/Wal-49/PBI0147 | Air-moisture capturing but low yield |
|  | 310 | Akbar |  | Zn fortified, high yielding cultivar |
|  | 332 | Qual2000 | Australian Source | Australian germplasm |
|  | 334 | Impala | TEAL/C93.8//PI 196101 | Australian germplasm |
|  | 331 | Orion | TATIARA/QAL2000 | Australian germplasm |
| *T. aestivum* hybrid | 307 | H1 (5) | AR-26-3-1 × 46-IBWSN-24 | High yielding |
|  | 308 | H2 (23) | AR-7-4 × 29 IBWSN-245 | High yielding |
|  | 309 | H3 (3) | AR-26-3-1 × Ujala-16 | High yielding |
| *T. durum* | 95 | D-2 | D-15728 | Best-performing durum in terms of yield traits |
|  | 96 | D-5 | D-15729 × Bellaroi/D-15729 | Good baking quality and high yielding durum |
|  | 97 | D-7 | D-16732 | Good baking quality |
|  | 336 | Saintly | KALKA S/2*TAMAROI | Australian germplasm |
|  | 337 | Bellaroi | 920405/920274 | Australian germplasm |
|  | 333 | Aurora | Australian Source | Australian germplasm |
|  | 335 | Jandaroi | 110780/111587 | Australian germplasm |
| *Triticale* | 90 | 1-white semi | Semi hooded Wal-49/mtc32 x Y158/mtc33/Y158 | High yielding |
|  | 91 | 2-Yellow | mtc32 x Y158//mtc33/Wal-49 | High yielding |
|  | 93 | 1-hooded | Wal-49/mtc32 x Y158/mtc32 | High yielding |

**Table S2: Mean square values for the leaf traits of 26 wheat genotypes under normal, drought and heat conditions**

| **Sources of variation** | **Df** | **LA** | **PH** | **GT** | **LR** | **FLat** | **FLT** | **FLL** | **FLW** | **FLA** |
| --- | --- | --- | --- | --- | --- | --- | --- | --- | --- | --- |
| **Replications** | 2 | 0.29 | 3.71 | 0.10 | 0.29 | 1.18 | 1.20 | 10.5 | 0.04 | 77.9 |
| **Treatments** | 2 | 13.04*** | 83.13*** | 0.09 | 3.41*** | 8.96** | 22.26*** | 1464.4*** | 1.56*** | 1973.6*** |
| **Genotypes** | 25 | 1.75*** | 57.47*** | 1.07*** | 0.88*** | 2.01*** | 2.39*** | 42.5*** | 0.23*** | 108.0*** |
| **Treatments * Genotypes** | 50 | 1.38*** | 14.05*** | 0.10*** | 0.90*** | 2.27*** | 0.97** | 28.3*** | 0.25*** | 89.8*** |
| **Residuals** | 153 | 0.33 | 3.29 | 0.20 | 0.36 | 0.30 | 0.34 | 4.4 | 0.04 | 14.8 |

Significant levels: *** < 0.001, ** < 0.01

LA: leaf angle, PH: prickle hairs, GT: groove type, LR: leaf rolling, FLat: flag leaf attitude, FLT: flag leaf twist, FLL: flag leaf length (cm), FLW: flag leaf width (cm), FLA: flag leaf area (cm^2^)

**Table S3: Mean square values for the plant traits of 26 wheat genotypes under normal, drought and heat conditions**

| **Sources of variation** | **Df** | **WT** | **ST** | **SD** | **L** | **PL** | **EL** | **PlH** | **DH** | **PT** | **DM** | **GFD** |
| --- | --- | --- | --- | --- | --- | --- | --- | --- | --- | --- | --- | --- |
| **Replications** | 2 | 0.08 | 0.25 | 0.43 | 18.3 | 16.5 | 0.5 | 5.8 | 16.9 | 4.0 | 24.2 | 70.7 |
| **Treatments** | 2 | 2.76*** | 3.75*** | 9.20*** | 2107.6** | 284.6*** | 71.8*** | 201.2*** | 75.6** | 108.0*** | 2311.9** | 1530.5*** |
| **Genotypes** | 25 | 0.23*** | 1.90*** | 1.40*** | 343.2*** | 100.9*** | 17.9*** | 777.9** | 29.0*** | 11.0*** | 82.23*** | 111.7*** |
| **Treatments * Genotypes** | 50 | 0.29*** | 1.49*** | 1.37*** | 194.1*** | 10.0*** | 0.8 | 6.7** | 12.7* | 7.6*** | 14.0 | 23.6 |
| **Residuals** | 153 | 0.03 | 0.20 | 0.09 | 33.9 | 4.4 | 0.9 | 3.9 | 8.4 | 3.6 | 17.3 | 25.6 |

Significant levels: *** < 0.001, ** < 0.01, * < 0.1

WT: wall thickness (mm), ST: stem stiffness, SD: stem diameter (mm), L: lodging (%), PL: peduncle length (cm), EL: ear length (cm), PlH: plant height (cm), DH: days to heading, PT: productive tillers, DM: days to maturity, GFD: grain filling duration (days)

**Table S4:** **Mean square values for the soil moisture content and physiological parameters of 26 wheat genotypes under normal, drought and heat conditions**

| **Sources of variation** | **Df** | **M** | **gs** | **P** | **T** | **WUE** |
| --- | --- | --- | --- | --- | --- | --- |
| **Replications** | 2 | 22.0 | 226.2 | 1.5 | 0.02 | 0.04 |
| **Treatments** | 2 | 2880.7** | 3688.6*** | 1006.0*** | 143.60** | 16.76*** |
| **Genotypes** | 25 | 174.7*** | 1984.3** | 20.8*** | 2.49*** | 1.84*** |
| **Treatments*Genotypes** | 50 | 112.5*** | 1229.1** | 12.1*** | 1.12** | 1.95*** |
| **Residuals** | 153 | 8.7 | 201.1 | 2.4 | 0.32 | 0.36 |

Significant levels: *** < 0.001, ** < 0.01, * < 0.1, * < 0.05

M: soil moisture content, gs: stomatal conductance (mmol H_2_O m^−2^ s^−1^), P: photosynthesis (µmol CO2 m^−2^ s^−1^), T: transpiration (mmol H_2_O m^−2^ s^−1^), WUE: photosynthetic water use efficiency (mmol CO_2_ mol^−1^ H_2_O)

**Table S5: Mean square values for the yield traits of 26 wheat genotypes under normal, drought and heat conditions**

| **Sources of variation** | **Df** | **EW** | **SW** | **HI/spike** | **S/S** | **S** | **BY/plot** | **GY/plot** | **HI/plot** |
| --- | --- | --- | --- | --- | --- | --- | --- | --- | --- |
| **Replications** | 2 | 0.010 | 0.01 | 3.3 | 3.2 | 63.9 | 7054 | 5884 | 46 |
| **Treatments** | 2 | 0.241 | 0.29*** | 50.1*** | 42.8*** | 50.5* | 1746962*** | 1529350*** | 8648*** |
| **Genotypes** | 25 | 3.628 | 2.69*** | 392.1*** | 48.5*** | 633.8*** | 7763 | 6929 | 119 |
| **Treatments*Genotypes** | 50 | 0.001*** | 0 | 3.7 | 9.2*** | 29.7*** | 9397** | 9046*** | 196*** |
| **Residuals** | 153 | 0 | 0 | 3.2 | 2.9 | 14.1 | 5108 | 4592 | 100 |

Significant levels: *** < 0.001, ** < 0.01, * < 0.1

EW: ear weight (g), SW: seed weight per spike (g), HI: harvest index (%), S/S: spikelet per spike, S: number of seeds per ear, BY/plot: biological yield per plot (g), GY/plot: grain yield per plot (g), HI/plot: harvest index per plot (%)


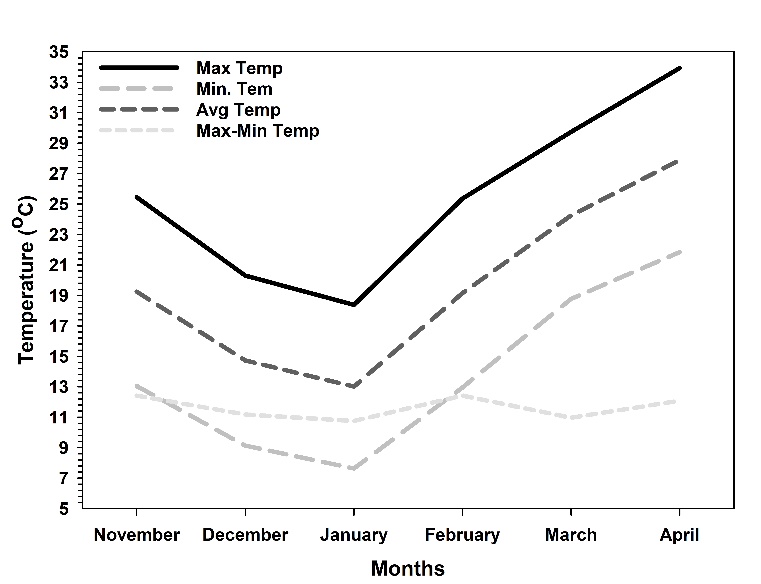

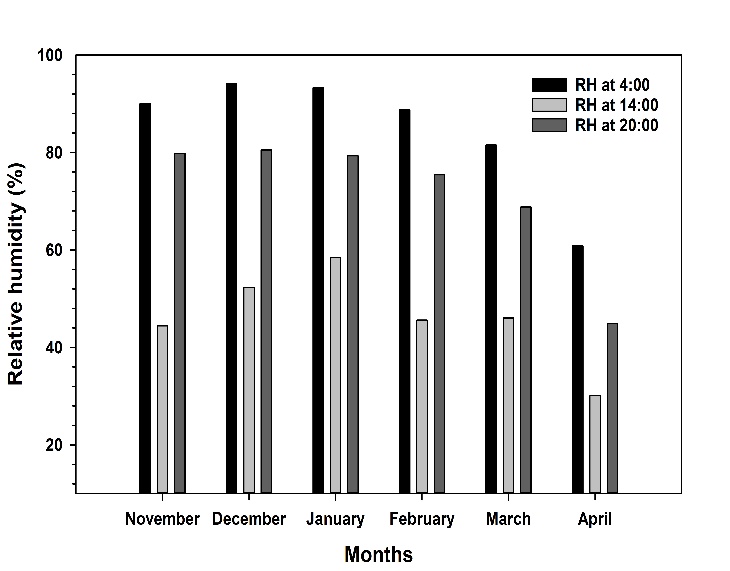


**b**

**a**

**Figure S1: Monthly average weather variables from November 15, 2020 to April 15-2021. a.** Average variables for maximum, minimum, average temperature, and difference of max to minimum temperature. **b.** Average relative humidity at 4:00, 14:00 and 20:00 hrs from November 2020 to April 2021. Temp: temperature, RH: relative humidity

**Figure S2: Daily average weather variables for the growing season 2020-21.** **a.** Daily average air temperature (maximum, minimum, average), wind speed, solar radiation, and rainfall during November 2020-April 2021. **b.** Daily average visibility range relative humidity during November 2020-April 2021. Slr: solar radiation, WS: windspeed, Air T_Avg: average air temperature, Air T_Max: maximum air temperature, Air T_min: minimum air temperature


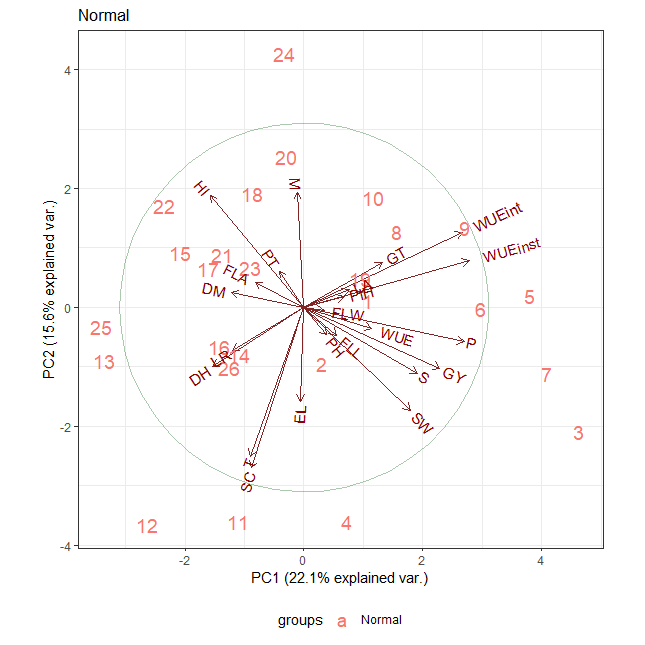

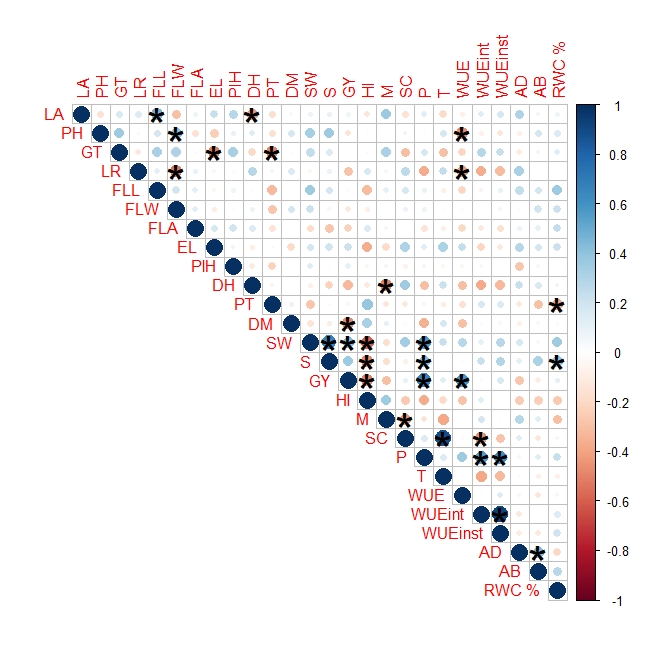

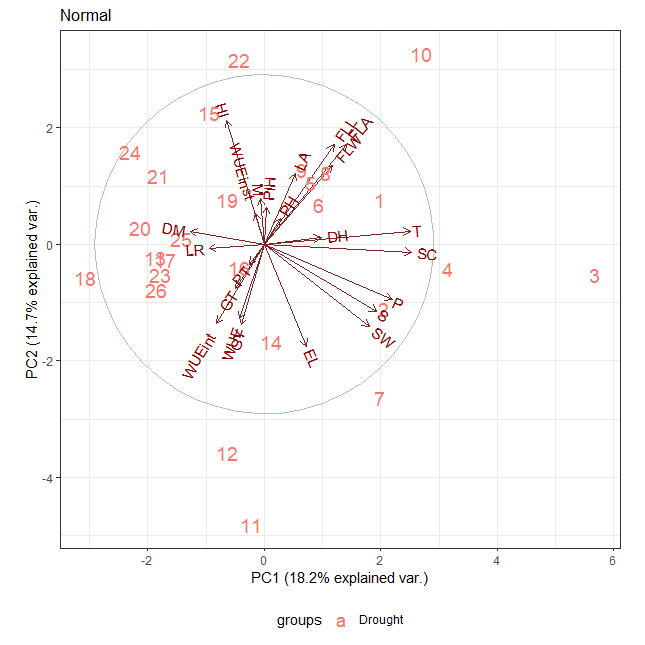

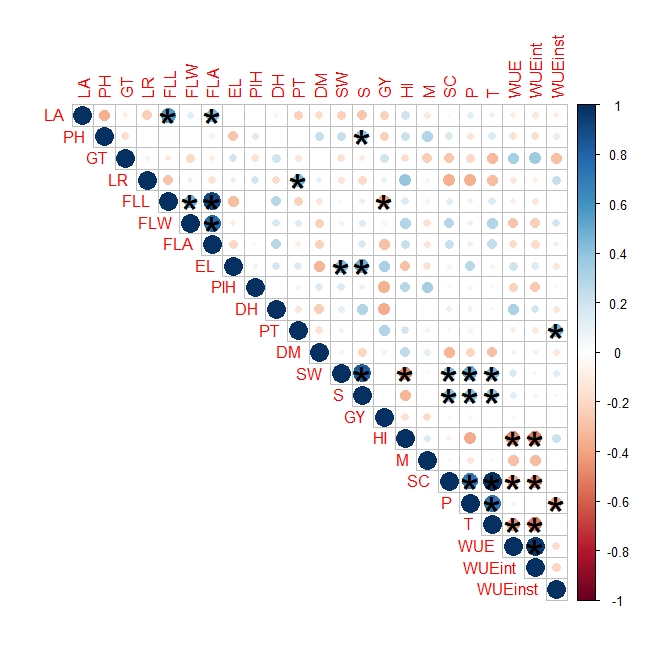


**e**

**d**

**c**

**b**

**a**


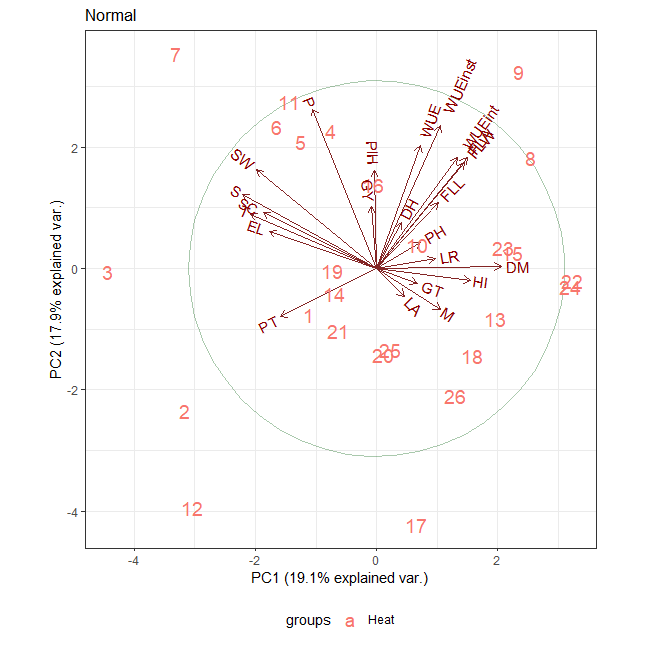

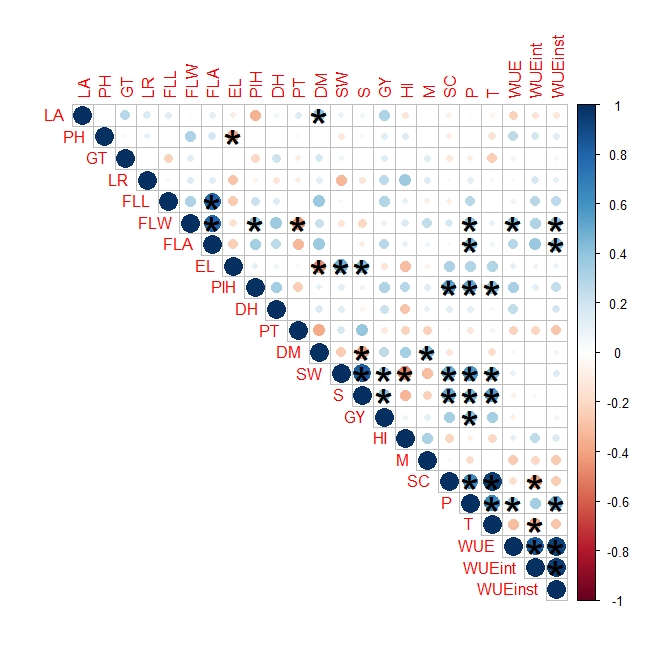


**f**

**Figure S3: Genotype-trait biplot and Pearson correlation analysis for the 26 wheat genotypes under normal (a & b, respectively), drought (c & d, respectively) and heat stress (e & f, respectively) conditions.** LA: leaf angle, PH: prickle hairs, GT: groove type, LR: leaf rolling, FLL: flag leaf length (cm), FLW: flag leaf width (cm), FLA: flag leaf area (cm^2^), EL: ear length (cm), PlH: plant height (cm), DH: days to heading, PT: number of productive tillers per plant, DM: days to maturity, SW: seed weight per ear (g), S: number of seeds per ear; GY: grain yield per plot (g), HI: harvest index, M: soil moisture content (%), SC: stomatal conductance (mmol H_2_O m^−2^ s^−1^), T: transpiration rate (mmol H_2_O m^−2^ s^−1^), photosynthetic water use efficiency (mmol CO_2_ mol^−1^ H_2_O), P: photosynthesis (µmol CO2 m^−2^ s^−1^), WUE_inst_: photosynthetic water use efficiency instantaneous, WUE_int_: photosynthetic water use efficiency intrinsic, AD: contact angle of the adaxial leaf surface (°C), AB: contact angle of the abaxial leaf surface (°C), RWC: relative water content (%)


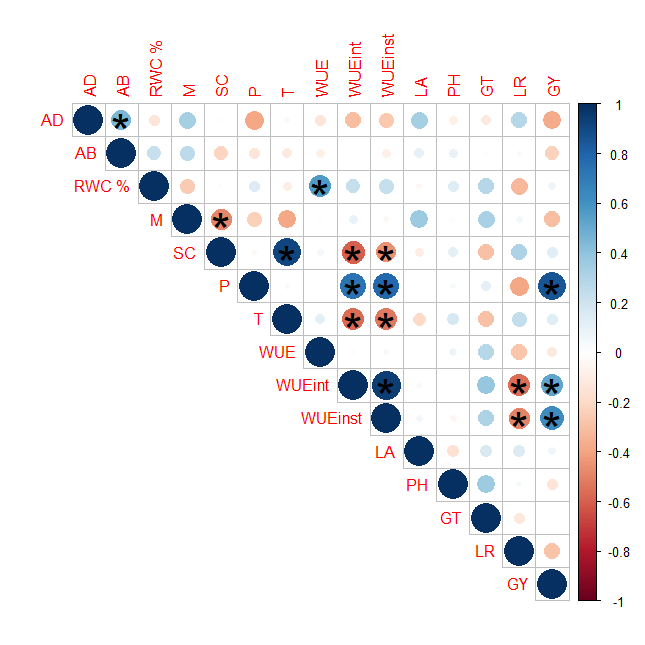


**Figure S4: Association of novel leaf traits, physiological traits, leaf wettability and relative water content under normal field conditions.** AD: contact angle on the adaxial leaf surface (°C), AB: contact angle on the abaxial leaf surface (°C), RWC: leaf relative water content (%), LA: leaf angle, PH: prickle hairs, GT: groove type, LR: leaf rolling, M: soil moisture content, SC: stomatal conductance (mmol H_2_O m^−2^ s^−1^), P: photosynthesis (µmol CO2 m^−2^ s^−1^), T: transpiration (mmol H_2_O m^−2^ s^−1^), WUE: photosynthetic water use efficiency (mmol CO_2_ mol^−1^ H_2_O), WUE_inst_: instantaneous water use efficiency, WUE_int_: intrinsic water use efficiency, GY: grain yield per plot (g)

**Figure S5:** Photosynthetic water efficiency of 26 genotypes of four different species including *T. aestivum*, *T. durum*, Triticale and *T. aestivum* hybrids under normal, drought and heat conditions


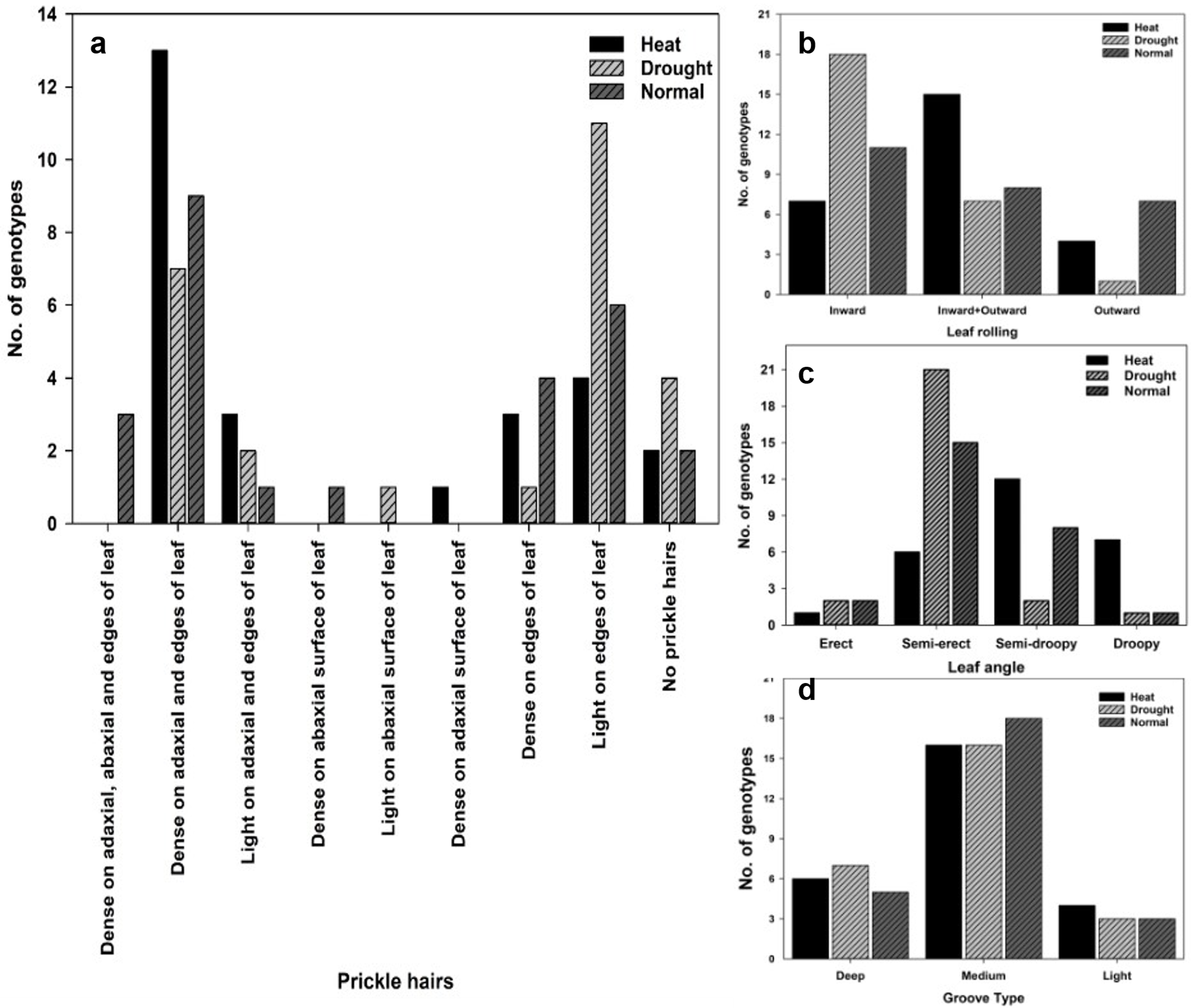


**Figure S6: Frequency distribution of 26 wheat genotypes for the four novel leaf traits under normal, drought and heat conditions.** **a.** Frequency distribution of 26 wheat genotypes for prickle hairs under normal, drought and heat conditions. **b.** Frequency distribution of 26 wheat genotypes for prickle hairs under normal, drought and heat conditions. **c.** Frequency distribution of 26 wheat genotypes for leaf angle under normal, drought and heat conditions. **d.** Frequency distribution of 26 wheat genotypes for groove type under normal, drought and heat conditions


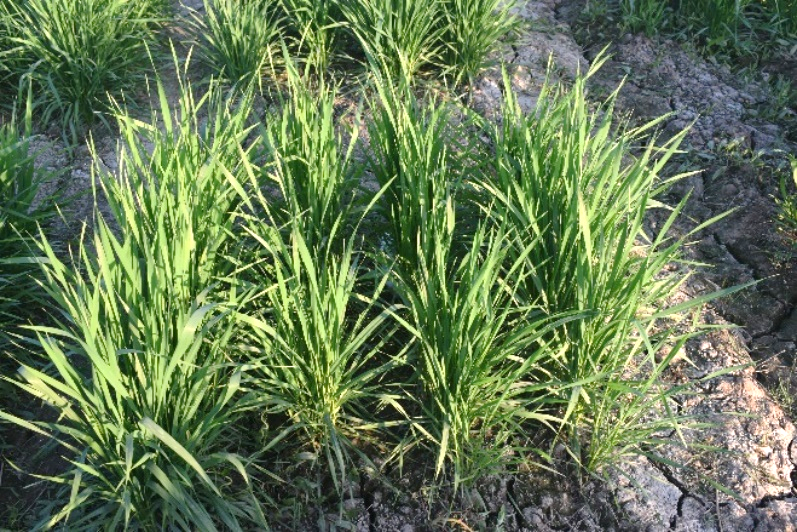

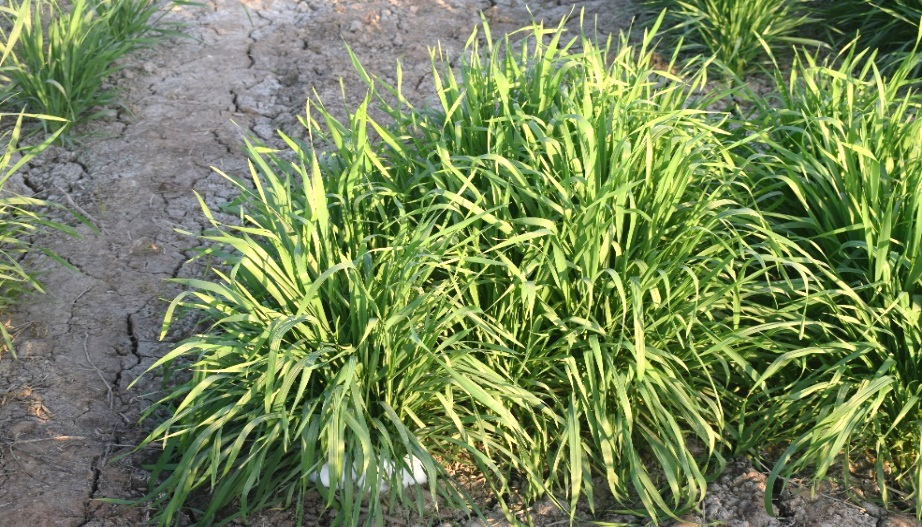


**Figure S7: Genotype 3 and 24 with contrasting leaf architecture used for evaluation of fog capturing under natural field conditions.**
